# From Molecular Mechanisms to Therapeutics: Understanding MicroRNA-21 in Cancer

**DOI:** 10.3390/cells11182791

**Published:** 2022-09-07

**Authors:** Jiho Rhim, Woosun Baek, Yoona Seo, Jong Heon Kim

**Affiliations:** 1 Cancer Molecular Biology Branch, Division of Cancer Biology, Research Institute, National Cancer Center, Goyang 10408, Korea; 2 Department of Cancer Biomedical Science, National Cancer Center, Graduate School of Cancer Science and Policy, Goyang 10408, Korea

**Keywords:** noncoding RNA, microRNA-21, cancer, biomarker, RNA therapeutics

## Abstract

MicroRNAs (miRNAs) are small noncoding RNAs that play an important role in regulating gene expression at a posttranscriptional level. As one of the first discovered oncogenic miRNAs, microRNA-21 (miR-21) has been highlighted for its critical role in cancers, such as glioblastoma, pancreatic adenocarcinoma, non-small cell lung cancer, and many others. MiR-21 targets many vital components in a wide range of cancers and acts on various cellular processes ranging from cancer stemness to cell death. Expression of miR-21 is elevated within cancer tissues and circulating miR-21 is readily detectable in biofluids, making it valuable as a cancer biomarker with significant potential for use in diagnosis and prognosis. Advances in RNA-based therapeutics have revealed additional avenues by which miR-21 can be utilized as a promising target in cancer. The purpose of this review is to outline the roles of miR-21 as a key modulator in various cancers and its potential as a therapeutic target.

## 1. Introduction

MicroRNAs (miRNAs) are small noncoding RNAs of approximately 22 nucleotides; they are intricately involved in a wide range of cellular functions, including cellular proliferation, apoptosis inhibition, neovascularization, DNA damage response, stress response, immunological response, and most importantly tumorigenic progression [1,2]. The human genome encodes more than 2000 mature miRNAs [3,4], and because miRNAs require only partial complementarity of 7–8 nucleotides (nt) for binding with the target mRNA, many coding transcripts harbor potential sites for miRNA-mediated gene expression regulation. As such, a single miRNA can target multiple cellular mRNA transcripts [2,5].

**Figure 1:**
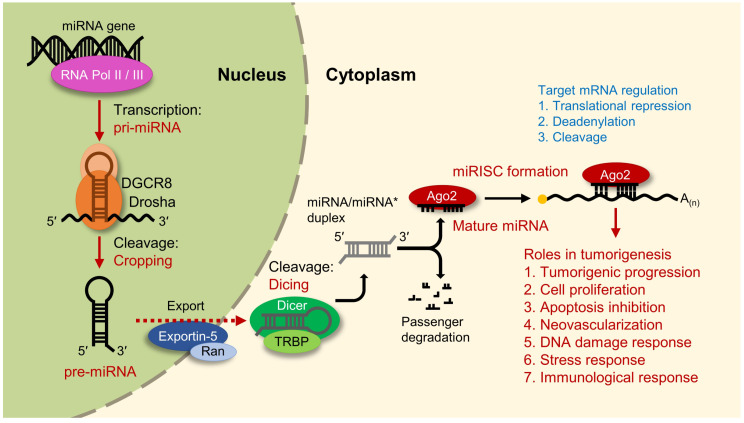
General miRNA biogenesis pathway and the related functions in cells; miRNA biogenesis begins with transcription by RNA polymerase II/III to create a pri-miRNA and is cleaved by Drosha/DGCR8 (cropping). The pre-miRNA is then exported to the cytoplasm via Exportin-5/Ran GTPase for further processing by Dicer/TRBP (dicing). Of this processed miRNA/miRNA* duplex, a “guide strand” is selected and loaded onto the Ago2 protein while the remaining passenger strand (miRNA*) is degraded. With the formation of a miRISC complex, target mRNA can be regulated and subsequently affect cellular and tumorigenic properties. pri-miRNA—primary microRNA, DGCR8—DiGeorge syndrome critical region 8, pre-miRNA—precursor microRNA, TRBP—transactivation response element RNA-binding protein, Ago2—argonaute protein 2, and miRISC—microRNA-induced silencing complex.

The biogenesis of an miRNA begins with the transcription of the initial encoding sequence by RNA polymerase II/III to produce a primary miRNA (pri-miRNA), which has a hairpin-like structure containing a 5′-7-methylguanosine (m^7^G) cap and poly(A) tail [6,7]. The pri-miRNA is processed by intronic excision through splicing (mirtron), or prominently by the RNase III endonuclease, Drosha, and the double-stranded RNA-binding protein, DiGeorge syndrome critical region 8 (DGCR8), which aids Drosha in the cleaving of the target site to release a 70 nt stem-loop precursor miRNA (pre-miRNA) with a 3′ overhang [8,9,10]. For further processing, the transcript is exported to the cytoplasm by Exportin-5 and the Ran GTPase, and undergoes an endoribonucleolytic reaction via Dicer, creating a 3′ overhang at both ends [11,12]. One strand is selected as the miRNA effector or “guide strand” based on the thermodynamic stability of the 5′ end of the duplex; the guide strand is loaded onto the argonaute protein 2 (Ago2) complex while the remaining strand, called the “passenger strand (miRNA*)”, is degraded [13] (Figure 1). This process forms general mature miRNAs.

Alternative forms called isomiRs, which contain variations in 5′ and mostly near the 3′ end, may arise through a shift in the Drosha/Dicer cleavage site or through Drosha-independent or Dicer-independent pathways. Terminal nucleotidyltransferases (TENTs) may also create variations by modifying miRNAs by extending the strand with several nucleotides at the 3′ ends. These additional nucleotides are eventually “trimmed” or “de-tailored” by poly (A)-specific ribonuclease (PARN) and other 3′-5′ exoribonucleases in a final maturation step [14,15,16].

In general, miRNA expression is perturbed in cancer, suggesting that there may be a link between aberrant miRNA expression and the disease. Furthermore, it is important to note the circumstances surrounding the regulation of the miRNA in question. Studies have highlighted how miRNA modulators can impact cancer. For example, the ubiquitin ligase, TRIM71, suppresses tumorigenesis by modulating let-7 in the Lin28B-let-7-HMGA2 signaling pathway [17,18]. Additionally, core miRNA processors and related factors, such as Drosha, Dicer, TRBP, and Ago2 are important in cancer progression [19]. In addition to these miRNA modulators and miRNAs, other noncoding RNAs subtypes have also been reported to regulate gene expression in various ways; these include circular RNAs, Piwi-interacting RNA, Y RNA, small nuclear RNA, small nucleolar RNA, vault RNA, tRNA-derived small RNA, and small interfering RNAs (Table 1). Accumulating evidence suggests that direct interactions between long noncoding RNAs (lncRNAs) and miRNAs can act as regulators in various diseases, especially in cancers, suggesting that the network of miRNA regulation is more intricate than previously understood [20,21]. Furthermore, although miRNAs have been primarily known to function as endogenous regulators of intracellular gene expression, the recent identification of miRNAs in a wide range of biological fluids suggests that they may also act systematically as signaling messengers, and thus could be useful as diagnostic or prognostic markers [22].

## 2. MicroRNA-21 and Its Role in Cancer

**Figure 2:**
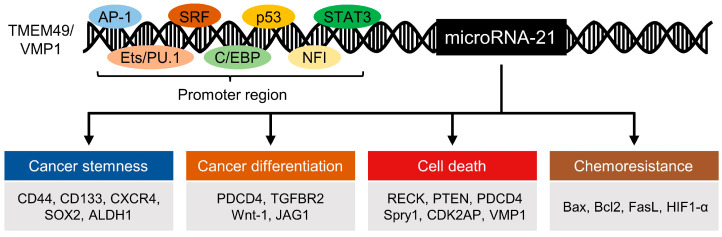
Epigenetic regulators of miR-21 and downstream targets of miR-21 in regards to cancer stemness, differentiation, cell death, and chemoresistance. Representative regulators of each cellular process are listed. TMEM49—transmembrane protein 49, VMP1—vacuole membrane protein 1, AP-1—activator protein 1, Ets/PU.1—Ets family transcription factor PU.1, SRF—serum response factor, C/EBP—CCAAT/enhancer binding protein, NFI—nuclear factor I, STAT3—signal transducer and activator of transcription 3, CXCR4—C-X-C chemokine receptor type 4, SOX2—sex-determining region Y-box, ALDH1—aldehyde dehydrogenase 1, PDCD4—programmed cell death 4, TGFBR2—transforming growth factor beta receptor 2, Wnt-1—wingless-integrated family member 1, JAG1—jagged canonical Notch ligand 1, RECK—reversion-inducing cysteine-rich protein with Kazal motifs, PTEN—phosphatase and tensin homolog, Spry1—sprouty RTK signaling antagonist 1, CDK2AP—cyclin-dependent kinase 2 associated protein, Bax—Bcl-associated X protein, Bcl2—B-cell lymphoma 2, FasL—Fas ligand, and HIF1-α—hypoxia-inducible factor 1-α.

Among the many miRNAs that have been associated with cancer progression, microRNA-21 (miR-21) was among the first to be identified as an oncogenic miRNA or “oncomiR”. Located on chromosome 17q23.2 in the intron of the transmembrane protein 49 (TMEM49)/vacuole membrane protein 1 (VMP1) gene, miR-21 was observed to have a unique, highly conserved promoter region that is activated by activation protein 1 (AP-1) in conjugation with the switch/sucrose non-fermentable (SWI/SNF) complex and Ets-related protein PU.1, CCAAT/enhancer binding protein (C/EBP), nuclear factor I (NFI), serum response factor (SRF), p53, and signal transducer and activator of transcription 3 (STAT3) [37,38]. Alteration of miR-21 expression has been linked to epigenetic factors and the dysregulation of transcriptional and posttranscriptional regulators, whether during biogenesis or through repression, resulting in oncogenic phenotypes (Figure 2).

Pivotal driver genes and their associated mutations, which commonly lie at the hub of many cellular pathways, can be found across different cancer types and may lead to dysregulation of or be affected by miR-21. Bailey and colleagues characterized common mutations across at least 15 or more cancer types which include TP53, PIK3CA, KRAS, phosphatase and tensin homolog (PTEN), and ARID1A [39]. Some of these mutations have been closely associated with miR-21 and its target proteins. In non-small cell lung cancer (NSCLC) containing R175H- and R248Q-mutant p53, miR-21 was observed to be upregulated, and the patients with elevated expression of p53 mutations and miR-21 had a lower overall survival rate. In addition, the knockdown of miR-21 led to lower mutant p53 mRNA levels [40]. Furthermore, induction of miR-21 appeared to mediate disease progression and metastasis in p53-deficient tumor keratinocytes [41]. This was also evident in KRAS mutation-driven cancers such as NSCLC and pancreatic ductal adenocarcinoma (PDAC). Inhibition of miR-21 in transgenic KRAS (G12D)/Trp53 null/Pdx1-cre (KPC) cell lines appeared to reduce proliferation, migration, and invasion compared to wild-type pancreatic epithelial cells [42]. Hatley and colleagues found that miR-21 overexpression in G12D-mutant KRAS mouse NSCLC models enhanced tumor formation, and subsequent deletion of miR-21 led to a significant decrease in total tumor mass in proportion to normal lung area [43]. Likewise, epidermal growth factor receptor (EGFR)-mutated lung cancer patients had considerably increased miR-21 expression compared to those without mutations [44]. In particular, EGFR is known to affect miRNA maturation through the posttranslational modification of Ago2, thus highlighting the important relationship between a cancer mutation and miR-21 status [45].

**Figure 3:**
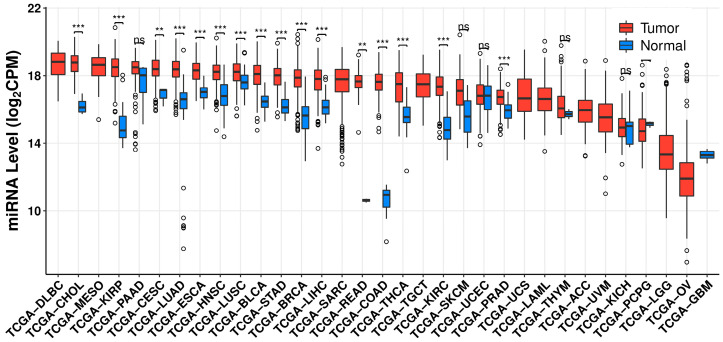
CancerMIRNome analysis of hsa-miR-21-5p expression in TCGA database demonstrating aberrant miR-21 expression across various cancer. Open circles indicate outliers. Some cancers (DLBC, MESO, SARC, TGCT, UCS, LAML, ACC, UVM, LGG, OV, and GBM) are incomparable in the dataset due to the lack of comparable tumor or normal samples. Significance was evaluated by CancerMIRNome via Wilcoxon rank-sum test. ** *p* < 0.01, *** *p* < 0.001, ns; not significant. DLBC—diffuse large B-cell lymphoma, CHOL—cholangiocarcinoma, MESO—mesothelioma, KIRP—kidney renal papillary cell carcinoma, PAAD—pancreatic adenocarcinoma, CESC—cervical squamous cell carcinoma and endocervical adenocarcinoma, LUAD—lung adenocarcinoma, ESCA—esophageal carcinoma, HNSC—head and neck squamous cell carcinoma, LUSC—lung squamous cell carcinoma, BLCA—bladder urothelial carcinoma, STAD—stomach adenocarcinoma, BRCA—breast invasive carcinoma, LIHC—liver hepatocellular carcinoma, SARC—sarcoma, READ—rectum adenocarcinoma, COAD—colon adenocarcinoma, THCA—thyroid carcinoma, TGCT—testicular germ cell tumor, KIRC—kidney renal clear cell carcinoma, SKCM—skin cutaneous melanoma, UCEC—uterine corpus endometrial carcinoma, PRAD—prostate adenocarcinoma, UCS—uterine carcinosarcoma, LAML—acute myeloid leukemia, THYM—thymoma, ACC—adrenocortical carcinoma, UVM—uveal melanoma, KICH—kidney chromophobe, PCPG—pheochromocytoma and paraganglioma, LGG—brain lower grade glioma, OV—ovarian serous cystadenocarcinoma, and GBM—glioblastoma multiforme.

Various independent studies determined that miR-21 is overexpressed in pancreatic adenocarcinoma, breast, and colorectal cancer (CRC) [46,47,48]. In a large-scale study using 540 human samples, miR-21 was found to be commonly overexpressed in lung, breast, stomach, prostate, colon, and pancreatic cancers [49]. MiR-21 was also found to be upregulated in glioblastoma [50,51]. Analysis of TCGA pan-cancer patient databases also reveals a similar trend across various cancers [52] (Figure 3).

Although the mechanisms linking miR-21 upregulation with other oncogenic factors have not yet been elucidated, various reports have indicated that miR-21 could drive cancer progression. In glioma, the β-catenin pathway was found to regulate miR-21 via STAT3, which is a well-known oncogenic transcription factor that promotes tumor growth and invasion [53,54]. MiR-21 also appears to be regulated by EGFR and the activation of β-catenin and AP-1. Upon upregulation, miR-21 regulates EGFR/Akt signaling by targeting the von Hippel–Lindau (VHL) and peroxisome proliferator-activated receptor alpha (PPAR-α) axes [55]. Furthermore, miR-21 upregulation appears to disrupt Ras/mitogen-activated protein kinase (MAPK) signaling via downregulation of Sprouty 2 (Spry2) leading to increased glioma malignancy [56]. MiR-21 also targets Spry2 in PDAC, resulting in the enhancement of epidermal growth factor-induced cell proliferation [57].

In NSCLC, miR-21 was found to repress the tumor suppressor PTEN, and promote growth and invasion phenotypes [58]. In breast cancer, programmed cell death 4 (PDCD4) was found to be directly regulated and repressed by miR-21 [59]. Additionally, miR-21 appeared to target and suppress the expression of tumor suppressor tropomyosin 1 (TPM1), mammary serine protease inhibitor Maspin, and PDCD4 in metastatic breast cancer, thereby affecting tumorigenicity, invasion, and metastasis [60].

Interestingly, Medina and colleagues reported that some cancers depend on one to several oncogenic genes including miR-21 called an “oncomiR addiction”. By using Tet-off and Cre recombinase technology to modulate miR-21 expression in a cross-bred mouse model, the authors showed that miR-21 upregulation led to and was necessary for the malignant phenotype of pre-B-cell lymphoma, which demonstrated this hypothesis. Importantly, the tumor regressed upon inactivation of miR-21, demonstrating the therapeutic validity of targeting miR-21 [61].

## 3. Regulation of MicroRNA-21 Biogenesis

Although many miRNAs are dysregulated in cancer, Thomson and colleagues demonstrated that pri-miRNA transcripts do not reflect the overexpression of their corresponding mature forms [62]. The authors suggested that the primary-to-precursor transcript turnover step in miRNA biogenesis may be disrupted in cancer. Extensive studies have indicated that RNA-regulatory proteins, such as heterogeneous nuclear ribonucleoprotein A1 (hnRNPA1), KH-type splicing regulatory protein (KSRP), and ribonuclease/angiogenin inhibitor 1 (RNH1) specifically interact with the Drosha microprocessor and facilitate miRNA maturation [63,64,65]. In a similar vein, members of the DEAD-box family of RNA helicases such as DDX5, DDX18, and DDX23 appeared to modulate miR-21 in various cancers [66,67,68]. The report of transforming growth factor beta (TGF-β) and bone morphogenetic protein (BMP) to induce miR-21 expression again highlights the importance of the Drosha microprocessor complex in miR-21 regulation. Moreover, ligand stimulation reportedly recruits the small mothers against decapentaplegic (SMAD) signal transducers, SMAD1/5 and SMAD2/3, with RNA helicase DDX5 to the pri-miR-21 resulting in the rapid maturation of miR-21 [69].

There may be yet-unknown miRNA regulation factors that contribute to tumor progression. IsomiRs of miR-21 have been found to be highly expressed in breast cancer and CRC, indicating that these noncanonical forms may contribute to the disease status [70,71]. Interestingly, it was recently reported that heterogeneous nuclear ribonucleoprotein C (hnRNPC) could directly interact with pri-miR-21 to induce an isoform shift of miR-21 in liver cancer cells. The resulting isomiR-21s were found to inhibit growth hormone receptors and promote tumorigenesis [72]. During the biogenesis of miR-21, it is commonly understood that the miR-21-5p strand acts as the guide strand and miR-21-3p is degraded as the passenger strand. However, recent studies showed that miR-21-3p is highly expressed in particular cancer cell lines, such as CRC cell lines and PC9 lung adenocarcinoma cells. Furthermore, isoforms of miR-21-3p were reported to show differential effects on cellular behaviors in CRC [73,74].

## 4. Regulation of Stemness Markers and Differentiation by MicroRNA-21

The term “cancer stem cells” (CSCs) refers to a small subpopulation of tumor cells that possess “stemness”, which comprises the stem cell-like abilities to self-renew and differentiate. These characteristics may be derived from the combined actions of various stemness-related transcriptional factors that are affected by miR-21 in various CSCs.

In PDAC cell lines, for example, miR-21 was reported to modulate key stemness factors, such as CD44, CD133, C-X-C chemokine receptor type 4 (CXCR4), and aldehyde dehydrogenase 1 (ALDH1), and miR-21 knockout was shown to significantly downregulate these factors [75]. Overexpression of miR-21 in CRC cells led to the downregulation of PDCD4 and the transforming growth factor beta receptor 2 (TGFBR2). Studies have shown that upregulation of TGFBR2 results in the downregulation of the key CSC markers, β-catenin, c-Myc, and cyclin-D, indicating that miR-21 may be involved in CRC differentiation [76,77]. MiR-21 was also found to suppress SOX2, a key stemness marker, in particular subsets of glioblastoma and mesenchymal stem cells [78,79].

Other independent studies revealed a number of ways in which miR-21 affects differentiation. The induction of Ras was found to dedifferentiate the thyroid-stimulating hormone (TSH)-dependent rat cell line FRTL5. Upregulation of miR-21 was observed during this process, indicating that miR-21 may also play a role in differentiation [50]. In human monocyte-derived dendritic cells, miR-21 and miR-34a could co-regulate differentiation through the repression of wingless-integrated family member 1 (Wnt-1) and jagged canonical Notch ligand 1(JAG1)-mediated signaling [80].

## 5. MicroRNA-21 in Cell Death

There are many forms of cell death, such as apoptosis, necroptosis, autophagy, and the more recently identified ferroptosis, and miR-21 has been found to be involved in almost all of them. The tumor suppressors PTEN and PDCD4, which play essential roles in the induction of apoptosis, are likely to be modulated by miR-21 via the PI3K/Akt/mTOR signaling pathway. Upon inhibition of miR-21, PTEN and PDCD4 were reportedly upregulated, leading to growth inhibition and apoptosis [59,81]. Sprouty1 (Spry1), an inhibitor of the Ras/MEK/ERK pathway, was also found to be targeted and inhibited by miR-21, leading to decreased apoptosis in cardiac fibroblasts [82]. In glioblastoma, inhibition of miR-21 leads to upregulation of the matrix metalloproteinase inhibitors, a reversion inducing cysteine-rich protein with Kazal motifs (RECK), and TIMP metallopeptidase inhibitor 3 (TIMP3), which decreases glioma motility and activates caspases [83]. In NSCLC, miR-21 appears to inhibit Akt expression and enhance apoptosis by interfering with the PI3K/Akt/NF-κB signaling pathway [84].

Although necroptosis is largely driven by receptor-interacting protein 1 (RIP1), RIP3, and mixed lineage kinase domain-like (MLKL), miR-21 was found to play an indirect role through the modulation of cyclin-dependent kinase 2-associated protein (CDK2AP) [85,86]. MiR-21 was also reported to suppress tumor suppressor genes (PTEN and FasL), and inhibition of miR-21 could reduce acute pancreatitis [87].

MiR-21 was observed to play significant roles in autophagy across various cancers including CRC, hepatocellular carcinoma (HCC), and glioma. In CRC, miR-21 was suggested to regulate autophagy by acting on the PTEN/Akt/transcription factor EB (TFEB) pathway. It inhibited VMP1 expression via phosphorylation of TFEB, which is important for autophagosome formation [88]. In HCC, sorafenib-resistant HCC cell lines were found to have lower levels of autophagy and elevated levels of miR-21. PTEN and Akt activation were also restricted in this system, indicating that miR-21 inhibits sorafenib-induced autophagy through the PI3K/Akt pathway [89]. In glioma, inhibition of miR-21 was found to increase the autophagy and radio-sensitivity of glioma cell lines, suggesting that miR-21 may contribute to radio-resistance by regulating autophagy [90].

Ferroptosis is a relatively new category of programmed cell death; it is defined by iron accumulation and lipid peroxidation, with subsequent oxidative cell death [91,92]. Elevated expression of miR-21 appeared to increase reactive oxygen species (ROS) levels across various cancers via its downstream targets STAT3, proline oxidase, and PDCD4, leading to oxidative stress. This indicated that miR-21 could play a role in ferroptosis [93]. Interestingly, delivery of miR-21-3p to melanoma cell lines was found to promote interferon-gamma (IFN-γ)-mediated ferroptosis by targeting thioredoxin reductase 1 and elevating ROS generation [94].

## 6. MicroRNA-21 in Chemoresistance

Although cancer treatment has improved greatly over the past decades, the eventual development of chemoresistance is a remaining major obstacle. miR-21 has also been shown to reduce the susceptibility of cancer cells to certain drugs. In an independent study, upregulation of miR-21 and the subsequent downregulation of PTEN was found to affect the sensitivity of MCF-7 breast cancer cells to doxorubicin. Conversely, this could be overcome through the application of an miR-21 inhibitor or the overexpression of PTEN [95]. MiR-21 was also found to contribute to cisplatin resistance in gastric cancer cells through the downregulation of PTEN and activation of the PI3K/Akt pathway [96]. In NSCLC, miR-21 silencing reversed multidrug resistance and subsequently reduced Akt phosphorylation to modulate the expression levels of the transcriptional factor E2F-1 and Twist. Thus, miR-21 appears to play a key role in drug resistance [97].

MiR-21 was linked to glioblastoma resistance against first-line treatment, temozolomide; this was found to act through regulation of the pro-apoptotic Bcl-2-associated X protein (Bax) and anti-apoptotic B-lymphoma 2 (Bcl-2) balance, and caspase-3 activity [98]. MiR-21 was shown to protect pancreatic cancer cells from apoptosis through the mediation of the FasL/Fas pathway [99], and to affect the radio-resistance of malignant glioma cells. Indeed, circulating miR-21 serves as a good prognostic indicator for chemoresistance in glioma patients [90,100]. Elevated miR-21 expression may indirectly confer chemoresistance through hypoxia, which is well known to aid in tumor immune evasion, facilitate the development of chemoresistance, and induce numerous factors that impact tumorigenesis. Under hypoxia, hypoxia-inducible factor HIF-1α was found to induce miR-21 and allow cancer cells to effectively bypass the hypoxia-associated reduction in proliferation by avoiding apoptosis [101].

## 7. MicroRNA-21 as a Biomarker in Cancer

MiRNAs are stably detectable in the plasma and serum, and thus may be developed as molecular biomarkers for minimally invasive tool in cancer diagnosis and prognosis [2,102]. MiRNAs can exist in biofluids as either a part of an extracellular vesicle (EV) or a non-vesicle-associated ribonucleoprotein complex and can be used to distinguish patho-logies at various stages of disease progression [103]. Given that miR-21 is highly expressed in a wide variety of cancers and linked to several oncogenic characteristics, it has been evaluated as a diagnostic and prognostic cancer biomarker [104,105,106,107,108,109].

Toiyama and colleagues found that serum miR-21 levels were significantly higher in patients with adenomas and CRC. Furthermore, high miR-21 expression in the serum and tissues could be associated with tumor size, metastasis, and patient survival [110]. Exosomal miRNA profiling of ovarian cancer patients showed that miR-21, miR-141, miR-200a, miR-200b, miR-200c, miR-203, miR-205, and miR-214 were significantly elevated and could be used to distinguish patients from healthy individuals [111]. MiR-21 may even be useful in rare cancers with few available biomarkers. In leptomeningeal metastasis (LM) patients, miR-21 expression was monitored using EVs extracted from cerebrospinal fluid (CSF) and found that aberrant miR-21 expression correlated with patient survival [112,113,114].

The potential use of miRNA as diagnostic or prognostic factors has become increasingly important as accessibility to big data continues to improve. Databases and tools such as Circulating MicroRNA Expression Profiling (CMEP) and CancerMIRNome can help researchers identify miRNAs of interest in a variety of cancers and give a preliminary picture of their clinical significance [52,115].

**Figure 4:**
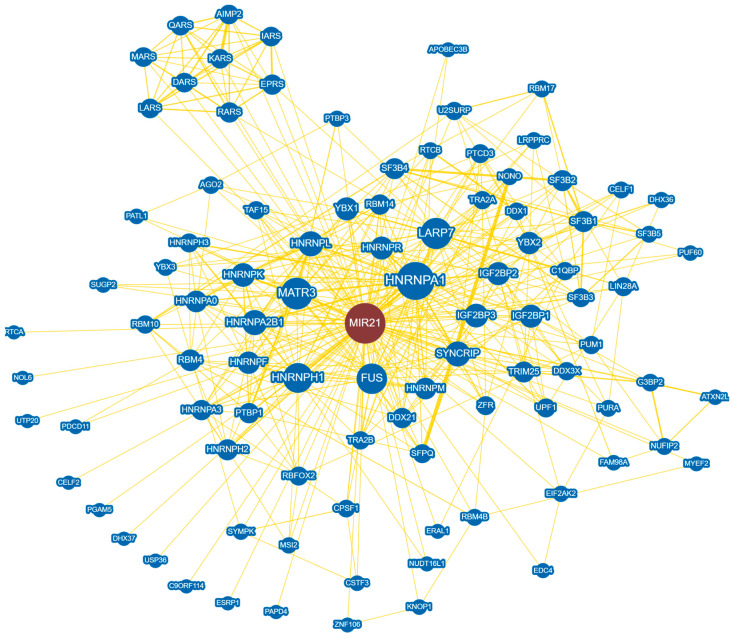
BioGRID protein interaction analysis of hsa-miR-21 interactome. In total, 96 protein interactions are displayed with an arbor layout in which related nodes are spaced closer together. Of the 96 protein interactors, 94 were found through high-throughput evidence, while 2 were found through low-throughput. AGO2—argonaute RISC catalytic component 2, AIMP2—aminoacyl tRNA synthetase complex-interacting multifunctional protein 2, APOBEC3B—apolipoprotein B mRNA editing enzyme catalytic subunit 3B, ATXN2L—ataxin-2-like protein, C1QBP—complement C1q binding protein, C9orf114—putative methyltransferase, CELF1/2—CUGBP Elav-like family member 1/2, CPSF1—cleavage and polyadenylation specific factor 1, CSTF3—cleavage stimulation factor subunit 3, DARS—aspartate-tRNA synthetase, DDX1/21/3X—DEAD-box helicase 1/21/3X, DHX36/37—DEAH-box helicase 36/37, EDC4—enhancer of mRNA decapping 4, EIF2AK2—eukaryotic translation initiation factor 2 alpha kinase 2, EPRS—glutamyl-prolyl-tRNA synthetase, ERAL1—Era-like 12S mitochondrial rRNA chaperone 1, ESRP1—epithelial splicing regulatory protein 1, FAM98A—family with sequence similarity 98 member A, FUS—FUS RNA binding protein, G3BP2—G3BP stress granule assembly factor 2, HNRNPA0/A1/A2B1/A3/F/H1/H2/H3/K/L/M/R—heterogeneous nuclear ribonucleoprotein A0/A1/A2B1/A3/F/H1/H2/H3/K/L/M/R, IARS—isoleucyl-tRNA synthetase, IGF2BP1/2/3—insulin-like growth factor 2 mRNA binding protein 1/2/3, KARS—lysyl-tRNA synthetase, KNOP1—lysine-rich nucleolar protein 1, LARP7—La ribonucleoprotein 7, LARS—leucyl-tRNA synthetase, LIN28A—lin-28 homologue A, LRPPRC—leucine-rich pentatricopeptide repeat containing, MARS—methionyl-tRNA synthetase, MATR3—matrin 3, MSI2—musashi RNA binding protein 2, MYEF2—myelin expression factor 2, NOL6—nucleolar protein 6, NONO—non-POU domain containing octamer binding, NUDT16L1—nudix hydrolase 16 like 1, NUFIP2—nuclear FMR1 interacting protein 2, PAPD4—PAP-associated domain-containing protein 4, PATL1—PAT1 homolog 1, PDCD11—programmed cell death 11, PGAM5—PGAM family member 5 mitochondrial serine/threonine protein phosphatase, PTBP1/3—polypyrimidine tract binding protein 1/3, PTCD3—pentatricopeptide repeat domain 3, PUF60—poly(U) binding splicing factor 60, PUM1—pumilio RNA binding family member 1, PURA—purine-rich element binding protein A, QARS—glutaminyl-tRNA synthetase, RARS—arginyl-tRNA synthetase, RBFOX2—RNA binding Fox-1 homolog 2, RBM10/14/17/4/4B—RNA binding motif protein 10/14/17/4/4B, RTCA/B—RNA 3′-terminal phosphate cyclase A/B, SF3B1/2/3/4/5—splicing factor 3b subunit 1/2/3/4/5, SFPQ—splicing factor proline/glutamine-rich, SUGP2—SURP and G-patch domain containing 2, SYMPK—symplekin, SYNCRIP—synaptotagmin binding cytoplasmic RNA interacting protein, TAF15—TATA-box binding protein associated factor 15, TRA2A/2B—transformer 2 alpha/2 beta homolog, TRIM25—tripartite motif containing 25, U2SURP—U2 snRNP-associated SURP domain containing, UPF1—UPF1 RNA helicase and ATPase, USP36—ubiquitin specific peptidase 36, UTP20—UTP20 small subunit processome component, YBX1/2/3—Y-box binding protein 1/2/3, ZFR—zinc finger RNA binding protein, and ZNF106—zinc finger protein 106.

The use of high-throughput visualization tools, such as BioGRID, could help researchers identify interactions between miR-21 and cellular protein partners, providing further insight into the role of miR-21 (Figure 4) [116,117]. In addition, predictive tools such as miRcode can be used to identify potential lncRNA-miRNA interactions, although it should be noted that these tools require more experimental validation [20,118]. It is still evident that there may be an underlying intricate network of mRNA-miRNA-lncRNA in disease progression that has yet to be explored. Improvements in a sensitive and live detection system for particular miRNAs such as miRDREL could also help illustrate a clearer picture of the action mechanism of oncomiRs such as miR-21 [119].

## 8. MicroRNA-21-Targeted RNA Therapeutics

Various studies have explored the possibility of targeting miR-21 with RNA-mediated therapeutics such as antisense oligonucleotides (ASO), locked nucleic acid (LNA)-anti-miRNAs, and most recently synthetic or natural circRNA, which act as miRNA sponges (Table 2).

ASOs are single-stranded RNAs that are complementary to a specific target RNA, especially miRNAs [133]. In pancreatic cancer cells, delivery of ASO-miR-21 suppressed the epithelial-mesenchymal transition and hindered proliferation. Furthermore, co-delivery of ASO-miR-21 with gemcitabine, a first-line drug for the treatment of locally advanced or metastatic pancreatic cancer acted synergistically in inducing apoptosis and growth inhibition [134,135]. Various in vivo studies utilizing miR-21 ASOs have yielded tumor growth inhibition in myeloma, HCC, glioblastoma multiforme (GBM), and breast cancer, demonstrating its therapeutic potential [136,137,138,139].

LNA technology “bridges” the 2′-O and 4′-C atoms in the ribose structure of RNA nucleotides, and is thus capable of improving the hybridization stability and specificity of anti-miRNAs [140,141]. In CRC, the application of LNA-anti-miR-21 was shown to effectively inhibit growth and induce apoptosis, demonstrating the clinical potential of using LNA for miRNA-targeting therapeutics [120]. Furthermore, fully LNA-modified phosphorothioate oligonucleotides or “tiny LNAs” (8-mer-long) were reported to target the seed regions of Ago2-bound miR-21 [142].

Chu and colleagues demonstrated that this technology could be applied in vivo through the systemic administration of LNA-miR-21 inhibitors to KPC mice expected to have low-grade pancreatic intraepithelial neoplasia 1 (PanIN-1) lesions. Mice that received LNA-miR-21 did not show disease progression, which demonstrates that LNA-miR-21 inhibitors could effectively interfere with malignant progression [42].

CircRNAs have been observed to play a variety of roles such as transcriptional regulation through interaction with U1 snRNP, or functional regulation through protein interactions as in the case of circFOXO3 [143,144]. Although other linear RNAs can also act as competing endogenous RNA (ceRNA), circRNAs are advantageous in structural stability and conservation which have led to growing interest in circRNAs in therapeutic applications [145]. In addition, circRNAs are capable of acting as ceRNA through miRNA sponging [146]. MiRNA sponges have also been shown to effectively regulate miR-21 in vitro [147,148]. Recently, both synthetic and natural circRNAs were found to act as effective miRNA sponges against miR-21 [149].

In NSCLC, natural circRNA (c0001287) sponged miR-21 to upregulate PTEN and thereby inhibit proliferation and metastasis [150]. Liu and colleagues created a synthetic circRNA to sponge miR-21 and found that the inhibition of miR-21 through this method could suppress gastric carcinoma cell proliferation by downregulating death domain-associated protein DAXX [131]. Interestingly, Muller and colleagues delivered polyethyleneimine (PEI) nanoparticles containing circular miR-21-5p decoys through intraperitoneal injection in subcutaneous xenograft mice and found significant potency in inhibiting tumor growth in vivo [151]. Thus, the importance of circRNA can be shown in its therapeutic potential in cancer treatment.

Recently, Wang and colleagues developed a method to simultaneously sponge miR-21 and deliver a chemotherapeutic drug. In this system, an oligonucleotide shell of a spherical nucleic acid of gold nanoparticles loaded with doxorubicin was shown to capture miR-21/miR-155, and thereby trigger the release of doxorubicin to enable tumor-specific chemotherapy [152].

## 9. Conclusions and Future Perspectives

Although approaches to identifying, utilizing, and inhibiting miR-21 have become more sophisticated over the past decade, many details remain unknown, such as how miR-21 matures, and what drives the formation of canonical versus noncanonical isomiR forms in certain cancers. Further understanding of these mechanisms and their consequences would provide more insight into the role of miR-21 in cancer.

It is now widely accepted that miRNA exist and function in both the intra- and extracellular environment. Few studies have examined the role (s) of miR-21 in intercellular communication in the tumor microenvironment, particularly in patients or patient-derived tumor models. Given the ability of miR-21 to target a wide variety of tumor suppressive and oncogenic pathways, such work could reveal additional functions for miR-21 in disease progression.

Regarding RNA-based therapeutics, several obstacles still limit the development of an effective anti-miR-21 for clinical use. Although multiple ASOs and LNAs have been developed to target miR-21, several of the relevant studies have been limited to in vitro experiments, meaning that the results may not reflect the nature of the tumor microenvironment or provide information on potential efficacy in the face of tumor heterogeneity. In addition, ASOs and LNAs have structural limitations that leave open questions regarding delivery, effectiveness, and prevention of unwanted side effects in the clinic.

Various miRNA-targeting RNA therapeutics are currently undergoing clinical trials, such as the miR-122 inhibitor miravirsen, the miR-16 inhibitor mesoMiR-1, and miR-21 inhibitor lademirsen. Thus, despite the above-described difficulties, RNA therapeutics are showing promise for clinical application [153].

Other methods of miR-21 inhibition can target specific steps in miRNA biogenesis. For example, the small-molecule inhibitors diazobenzene and estradiol, have been used to target specific steps in miRNA biogenesis such as transcription [154,155,156]. In other efforts, the miRNA structure has been directly targeted by using small molecules to bind to the G-hairpin of the hTERT-G-quadruplex-forming sequence and thereby downregulate expression. This strategy achieved strong anticancer effects in mice [157]. However, similar to the above-described RNA therapeutics, there remain issues with delivery, effectiveness, and toxicity, and further research would be needed to support the development of effective and specific miR-21 targeting inhibitors.

Current evidence demonstrates that miR-21 exerts immense influence on cancer progression, including strong effects on cancer cell proliferation, stemness, apoptosis, and chemoresistance. In addition, circulating miR-21 can not only be used as a diagnostic tool but also as a targetable functional component of systemic intercellular communication in cancers such as GBM and LM [108,112]. Although the ability to predict interactions and create therapeutic avenues for inhibiting miR-21 has advanced significantly in the past decade, complications related to cancer heterogeneity and the intricate web of the microenvironment itself have become increasingly apparent. Furthermore, evidence points to how treatment must take into account the underlying miRNA-mRNA, cellular protein modulators, and ncRNA network to develop effective therapeutics that can overcome recent limitations in cancer treatment. Our understanding of miR-21 and cancer has grown in the past decade, and soon we may be able to provide an answer in the form of an effective and safe RNA-based treatment for clinical applications.

## Figures and Tables

**Table 1:** Different types of existing noncoding RNA.

Subtype	Abbreviation	Size (nt)	Description	References
Circular RNA	circRNA	100 ~ ≥4000	Functions through interaction with RNA and RNA binding proteins; modulating stability and regulating gene transcription	[23,24,25]
Enhancer RNA	eRNA	≤2000	Transcribed upon activation of enhancers and affects efficiency of enhancer activation and gene transcription	[26,27]
Piwi-interacting RNA	piRNA	26–31	Forms RNA-protein complexes with Piwi-subfamily proteins and involving in epigenetic and post- transcriptional silencing of transposable elements	[28]
Small interfering RNA	siRNA	21–25	Plays a role in RNA interference through silencing of transposons and mediating transcriptional gene silencing	[29]
Small nuclear RNA	snRNA	~150	Primarily functions in processing of pre-mRNA or heterogeneous nuclear RNA (hnRNA) in the nucleus	[30]
Small nucleolar RNA	snoRNA	~70–250	Primarily guide chemical modification and processing of other RNAs and also function as miRNA	[31]
Telomerase RNA component	TERC	150–1300	Helps constitute telomerase activity in tandem with TERT and acts as a template for telomere replication	[32]
tRNA-derived small RNA	tsRNA	18–40	Regulates gene expression similarly to miRNA- mediated RNA silencing mechanism via interacting with Ago proteins	[33]
Vault RNA	vtRNA	86–141	Associated with drug resistance and regulates expression through miRNA-like RNA silencing mechanism via interacting with Ago proteins	[34]
Y RNA	Y RNA	84–113	Components of Ro60 ribonucleoprotein and involved in various cellular processes such as DNA replication and RNA quality control	[35,36]

**Table 2:** Therapeutic approaches of miR-21 targeting in cancers.

Type	Target Cancers	Cell Lines & Experimental Models	Phenotypes	References
LNA	Colorectal adenocarcinoma	LS174T	Proliferation inhibited; apoptosis enhanced	[120]
	Melanoma	B16F10; C57BL/6 mice	Tumor growth and volume inhibited; apoptosis enhanced	[121]
	Glioblastoma	U87MG; Orthotopic xenograft in athymic nude mice	Tumor growth inhibited; apoptosis enhanced	[122,123]
	Non-small cell lung cancer	A549; Female nude mice	Drug sensitization; tumor growth inhibited; apoptosis enhanced	[124]
	Breast cancer	MCF-7	Proliferation inhibited	[59]
ASO	Laryngeal squamous cell carcinoma	Hep-2; BALB/c nude mice	Tumor growth and proliferation inhibited; invasiveness decreased; cell cycle arrest; apoptosis enhanced	[125]
	Non-small cell lung cancer	PC9; Female BALB/c nude mice	Proliferation inhibited; apoptosis enhanced; tumor growth inhibited	[126]
	Glioblastoma multiforme	LN229; U251MG; U373MG; T98G	Drug sensitization; cell viability decreased	[127,128]
	Hepatocellular carcinoma	Huh7; HepG2	Migration and invasiveness decreased	[129]
	Breast phyllode tumor	Patient-derived breast stromal cells; Female nude mice	Proliferation inhibited and invasion decreased	[130]
CircRNA	Gastric carcinoma	NCI-N87; AGS; MKN28	Proliferation inhibited	[131]
	Lung cancer	L132, A549; LL2; 3D multicellular spheroids	Proliferation inhibited; migration decreased; apoptosis enhanced	[132]

## Data Availability

The data (Figure 3 and Figure 4) presented in this study are openly available in CancerMIRNome at https://doi.org/10.1093/nar/gkab784 (accessed on 31 August 2022) [52] and in the BioGRID database at https://doi.org/10.1002/pro.3978 (accessed on 31 August 2022) [116].

## Abbreviations

Ago2, argonaute protein 2; ALDH1, aldehyde dehydrogenase 1; AP-1, activator protein 1; ARID1A, AT-rich interaction domain 1A; ASO, antisense oligonucleotide; Bax, Bcl-associated X protein; Bcl-2, B-cell lymphoma 2; BMP, bone morphogenetic protein; CDK2AP, cyclin dependent kinase 2 associated protein; C/EBP, CCAAT/enhancer binding protein; ceRNA, competing endogenous RNA; circRNA, circular RNA; CMEP, circulating microRNA expression profiling; CRC, colorectal cancer; CSC, cancer stem cell; CSF, cerebrospinal fluid; CXCR4, C-X-C chemokine receptor type 4; DDX5/18/23, DEAD-box protein 5/18/23; DGCR8, DiGeorge syndrome critical region 8; EGFR, epidermal growth factor receptor; EV, extracellular vesicle; Fas, Fas cell surface death receptor; FasL, Fas ligand; FRTL5, Fischer rate thyroid cell line; GHR, growth hormone receptor; GBM, glioblastoma multiforme; HCC, hepatocellular carcinoma; HMGA2, high mobility group AT-hook 2; HIF-1α, hypoxia-inducible factor 1α; hnRNPA1, heterogeneous nuclear ribonucleoprotein A1; hnRNPC, heterogeneous nuclear ribonucleoprotein C; IFN-γ, interferon-gamma; JAG1, jagged canonical Notch ligand 1; KPC, KRAS (G12D)/Trp53 null/Pdx1-cre; KRAS, Kirsten rat sarcoma viral oncogene homologue; KSRP, KH-type splicing regulatory protein; LM, leptomeningeal metastasis; LNA, locked nucleic acid; lncRNA, long noncoding RNA; m^7^G, 5′-7-methylguanosine; Maspin, mammary serine protease inhibitor; MAPK, mitogen-activated protein kinase; miR-21, microRNA-21; miRDREL, miRNA-controlled dual reporter-expressing lentivirus; miRNA, microRNA; MLKL, mixed lineage kinase domain; mTOR, mammalian target of rapamycin; NFI, nuclear factor I; NSCLC, non-small cell lung cancer; nt, nucleotides; oncomiR, oncogenic microRNA; PARN, poly(A)-specific ribonuclease; PanIN-1, pancreatic intraepithelial neoplasia 1; PDAC, pancreatic ductal adenocarcinoma; PDCD4, programmed cell death 4; PEI, polyethyleneimine; PI3K, phosphoinositide 3-kinase; PIK3CA, phosphatidylinositol-4,5-biphosphate 3-kinase catalytic subunit alpha; PPAR-α, peroxisome proliferator activated receptor alpha; pre-miRNA, precursor microRNA; pri-miRNA, primary microRNA; PTEN, phosphatase and tensin homolog; RECK, reversion inducing cysteine-rich protein with Kazal motifs; RIP, receptor interacting protein; RNH1, ribonuclease/angiogenin inhibitor 1; ROS, reactive oxygen species; SMAD, small mothers against decapentaplegic; SOX2, sex determining region Y-box 2; Spry1/2, Sprouty RTK signaling antagonist 1/2; SRF, serum response factor; STAT3, signal transducer and activator of transcription 3; SWI/SNF, switch/sucrose non-fermentable; TRBP, transactivation response element RNA-binding protein; TENT, terminal nucleotidyltransferase; TFEB, transcription factor EB; TGF-β, transforming growth factor beta; TGFBR2, transforming growth factor beta receptor 2; TIMP3, TIMP metallopeptidase inhibitor 3; TMEM49, transmembrane protein 49; TNF-α, tumor necrosis factor alpha; TP53, tumor protein p53; TPM1, tropomyosin alpha-1; TRIM71, tripartite motif containing 71; TSH, thyroid-stimulating hormone; VHL, von Hippel-Lindau; VMP1, vacuole membrane protein 1; Wnt-1, wingless-integrated family member 1.
